# Donor Genotype in the Interleukin-7 Receptor α-Chain Predicts Risk of Graft-versus-Host Disease and Cytomegalovirus Infection after Allogeneic Hematopoietic Stem Cell Transplantation

**DOI:** 10.3389/fimmu.2018.00109

**Published:** 2018-02-02

**Authors:** Katrine Kielsen, Christian Enevold, Carsten Heilmann, Henrik Sengeløv, Anders Elm Pedersen, Lars P. Ryder, Klaus Müller

**Affiliations:** ^1^Institute for Inflammation Research, Department of Rheumatology and Spine Disease, Copenhagen University Hospital Rigshospitalet, Copenhagen, Denmark; ^2^Haematopoietic Stem Cell Transplantation and Primary Immune Deficiency, Department of Pediatrics and Adolescent Medicine, Copenhagen University Hospital Rigshospitalet, Copenhagen, Denmark; ^3^Department of Hematology, Copenhagen University Hospital Rigshospitalet, Copenhagen, Denmark; ^4^Department of Immunology and Microbiology, University of Copenhagen, Copenhagen, Denmark; ^5^Department of Odontology, University of Copenhagen, Copenhagen, Denmark; ^6^The Tissue Typing Laboratory, Department of Clinical Immunology, Copenhagen University Hospital Rigshospitalet, Copenhagen, Denmark

**Keywords:** interleukin-7 receptor, single nucleotide polymorphisms, allogeneic hematopoietic stem cell transplantation, graft-versus-host disease, T cell reconstitution, cytomegalovirus infection

## Abstract

The efficacy of allogeneic hematopoietic stem cell transplantation (HSCT) is challenged by acute and chronic graft-versus-host disease (aGVHD and cGVHD) and viral infections due to long-lasting immunodeficiency. Interleukin-7 (IL-7) is a cytokine essential for *de novo* T cell generation in thymus and peripheral T cell homeostasis. In this study, we investigated the impact of the single nucleotide polymorphism *rs6897932* in the IL-7 receptor α-chain (IL-7Rα) which has previously been associated with several autoimmune diseases. We included 460 patients undergoing allogeneic HSCT after a myeloablative conditioning. Patients had a median age of 26.3 years (0.3–67.0 years), and 372 (80.9%) underwent HSCT for malignant diseases. Donors were matched sibling donors (*n* = 147), matched unrelated donors (*n* = 244) or mismatched unrelated donors (*n* = 69), and the stem cell source were either bone marrow (*n* = 329) or peripheral blood (*n* = 131). DNA from donors was genotyped for the IL-7Rα single nucleotide polymorphism (SNP) *rs6897932* using an allele-specific primer extension assay (CC: *n* = 252, CT: *n* = 178, TT: *n* = 30). The donor T allele was associated with a higher risk of grades III–IV aGVHD (HR = 2.0, 95% CI = 1.1–3.8, *P* = 0.034) and with significantly increased risk of extensive cGVHD (HR = 2.0, 95% CI = 1.1–3.6, *P* = 0.025) after adjustment for potential risk factors. In addition, the TT genotype was associated with a higher risk of cytomegalovirus (CMV) infection post-transplant (HR = 2.4, 95% CI = 1.2–4.3, *P* = 0.0068). Numbers of T cells were significantly higher on day +60 in patients receiving a *rs6897932* TT graft (CD3+: 109% increase, *P* = 0.0096; CD4+: 64% increase, *P* = 0.038; CD8+: 133% increase, *P* = 0.011). Donor heterozygosity for the T allele was associated with inferior overall survival (HR = 1.7, 95% CI = 1.2–2.3, *P* = 0.0027) and increased treatment-related mortality (HR = 2.3, 95% CI = 1.3–4.0, *P* = 0.0047), but was not associated with the risk of relapse (*P* = 0.35). In conclusion, the IL-7Rα *rs6897932* genotype of the donor is predictive of aGVHD and cGVHD, CMV infection, and mortality following HSCT. These findings indicate that IL-7Rα SNP typing of donors may optimize donor selection and facilitate individualization of treatment in order to limit treatment-related complications.

## Introduction

Allogeneic hematopoietic stem cell transplantation (HSCT) is a treatment of high-risk leukemia and a number of benign hematological disorders. In the treatment of leukemia, the outcome of HSCT is based on an immune-mediated cytotoxic attack on the malignant cells and persisting immune surveillance, also known as the graft-versus-leukemia effect. However, the success of HSCT is limited by long-lasting T cell dysfunction with risk of severe infections and development of graft-versus-host disease (GVHD), both contributing significantly to non-relapse mortality (1–3). More detailed insight into the mechanisms of T cell reconstitution and prognostic markers is essential to limit morbidity and mortality after HSCT.

Interleukin-7 (IL-7) is a hematopoietic cytokine essential for *de novo* T cell development in the thymus and homeostatic peripheral expansion of T cells (4–6). IL-7 signals through the IL-7 receptor (IL-7R), a heterodimer consisting of the common γ-chain (CD132) and the high-affinity IL-7R α-chain (IL-7Rα, CD127) (7). The IL-7Rα-chain is also used by Thymic Stromal Lymphopoetin, a cytokine promoting TH2 differentiation and Treg induction, and involved in allergic inflammation and autoimmunity (8–12).

Interleukin-7 receptor α-chain is expressed on lymphocyte progenitors and on naïve and memory T cells, and its expression is strictly regulated during the different developmental stages of T cells with the highest expression on naïve T cells, a lower expression on memory T cells, and downregulation of IL-7Rα upon development into effector T cells (6, 13). The critical role of the IL-7 pathway for human T cell homeostasis is illustrated by the fact that absence of a functioning IL-7Rα leads to severe combined immunodeficiency with a T-B + NK + phenotype (14), while somatic gain-of-function mutations in IL-7Rα may cause T- as well as B-cell acute lymphoblastic leukemia (15, 16).

Single nucleotide polymorphisms (SNPs) in the exons of the IL-7Rα, which give rise to non-conservative amino-acid substitutions, have been associated with several chronic inflammatory diseases. The *rs6897932* SNP in the transmembrane region of the IL-7Rα increases the risk of developing multiple sclerosis, ulcerative colitis, and sarcoidosis (17–20). In allogeneic HSCT, donor genotypes of SNPs influencing the structure of the extracellular part of IL-7Rα have been associated with non-relapse mortality after allogeneic HSCT, in contrast to recipient genotypes that were not associated with outcomes (21–23).

In this study, we show that the donor genotype in IL-7Rα *rs6897932* influences the rate of immune reconstitution after allogeneic HSCT with impact on infections as well as acute GVHD (aGVHD) and chronic GVHD (cGVHD).

## Materials and Methods

### Patient Population

We retrospectively studied patients undergoing allogeneic transplantation at the national HSCT center at Copenhagen University Hospital Rigshospitalet, Denmark, from 2004 to 2014. Inclusion criteria were first allogeneic HSCT, myeloablative conditioning (24), a matched sibling donor or an unrelated donor, and the use of bone marrow or peripheral blood as stem cell source.

Five-hundred twelve patients fulfilled the inclusion criteria. Deposited donor blood samples were available for 471 of these, and a donor SNP could be assigned for 460 patients (89.8%), which were all included in the study. The included patients did not differ significantly from non-participants in terms of age, diagnosis, donor, conditioning regimen, graft type, cell dose/kilogram, pre-transplant Karnofsky score, sex-mismatch, or cytomegalovirus (CMV) antibody status.

The study protocol was approved by the ethics committee of the Capital Region of Denmark (#H-15006001), and written informed consent was obtained from all patients and/or their legal guardians.

### Patient Characteristics

The study included 153 children and 307 adults with a median age of 26.3 years (range 0.3–63.0 years). Diagnosis was acute myeloid leukemia (*n* = 136), acute lymphoblastic leukemia (*n* = 118), myelodysplastic syndrome (*n* = 51), chronic myeloid leukemia (*n* = 39), other malignancies (*n* = 28), or benign diseases (*n* = 88, including 46 severe aplastic anemia and 24 primary immunodeficiencies). Donors were either fully human leukocyte antigen-A, -B, -C, -DR, and -DQ allele-matched sibling donors (*n* = 147), matched unrelated donors (10/10 match, *n* = 244), or mismatched unrelated donors (9/10 or 8/10 match, *n* = 69). Bone marrow (*n* = 329) or G-CFS mobilized peripheral blood stem cells (*n* = 131) were used as stem cell source, and allografts were T cell replete. Conditioning regimens consisted of total body irradiation (TBI) plus cyclophosphamide or etoposide (*n* = 293), cyclophosphamide plus busulphane (*n* = 107), or other types of chemotherapy-based conditioning (*n* = 60). Conditioning included anti-thymocyte globulin (ATG) in 179 patients transplanted with an unrelated donor. GVHD prophylaxis consisted of Cyclosporine A plus methotrexate for 90% of patients. All patients were monitored weekly by PCR for viral infections (reactivation or primary infection) with CMV and Epstein–Barr virus (EBV) until day +90 post-HSCT and subsequently every second week. In case of increasing viral load, pre-emptive treatment with anti-viral medication [(Val)ganciclovir for CMV infection and Rituximab for EBV infection] was commenced along with tapering of immunosuppression.

### Detection of IL-7Rα SNPs

Blood from donors were collected before HSCT and stored at −20°C. Genomic DNA extraction was performed using Maxwell™ 16 Blood DNA Purification Kit (Promega Biotech AB, Nacka, Sweden) as described by the manufacturer.

DNA was genotyped for *rs6897932* using a previously described multiplex bead-based assay (25). In brief, allele-specific primers were labeled in a primer extension using polymerase chain reaction-amplified SNP-sites as their target regions. The labeled primers were then hybridized to MicroPlex-xTAG beadsets for detection and counting on the Luminex platform (Luminex Corporation, Austin, TX, USA). We also included primers for the sex-specific amelogenin-gene (“AMELX” and “AMELY,” respectively) to be able to define the sex of the donor as a quality control (26).

All donor samples were blinded to the technicians performing the analyzes. The IL-7Rα SNP calling rates were 99.4%, and 10% of samples were genotyped twice without discordance. Eight samples were excluded due to mismatch between sex according to sex determined by the amelogenin-gene and known donor sex.

### Immunological Parameters

Absolute lymphocyte counts were measured as part of the clinical routine by particle counting using Sysmex XN flow cytometry. Total immunoglobulins (IgM, IgG, and IgA) were measured with turbidimetry using Cobas 8000, module c502.

T and B cells were counted 12 months after HSCT, and in addition measured after 1, 2, 3, and 6 months in patients undergoing HSCT from 2008 to 2014 (*n* = 283, 62%). Peripheral blood samples were analyzed directly in a single-platform no-lyse-no-wash flow cytometry procedure. EDTA-anti-coagulated blood were incubated in Trucount tubes (Becton, Dickinson & Company, Albertslund, Denmark) for quantification of lymphocyte subsets, and a panel of monoclonal antibodies (CD3-PerCP, CD3-FITC, CD4-FITC, CD8-PE, CD45-PerCP, and CD19-PE, all from BD) on a FC500 flow cytometer (Beckman Coulter, Copenhagen, Denmark). Lymphocytes were gated based on forward scatter and side scatter characteristics. Lymphocyte subsets were identified as CD3+ T cells, CD4+ T cells, CD8+ T cells, and CD45+ CD19+ B cells. The laboratory participates in the quality assurance program by the National External Quality Assessment Site (NEQAS).

### Statistical Analysis

Kaplan–Meier estimates with log-rank tests were applied as an initial non-parametric analysis of the risk of complications and mortality. Next, a cause-specific Cox regression model was used to estimate the risk of aGVHD, cGVHD, viral reactivation, and time to lymphocyte recovery. Overall survival was analyzed further using Cox regression, while treatment-related mortality (TRM) and relapse were estimated using Fine–Gray competing risk regression model. For all analyzes, transplant-related characteristics that are main risk factors for the specific transplant-related complications were included in the multivariable model for this outcome as indicated under results.

Linear regression analyzes were used to analyze associations with cell counts and immunoglobulin levels after 1 year. Longitudinal analysis of T cell counts were performed with a linear mixed model with random slope over time since transplantation and random intercept by patient for measurements from all patients without truncation due to death, relapse, retransplantation, or donor lymphocyte infusion within the first 360 days. All cell counts were log10-transformed; and measurements equal to 0 (3.2%) were changed to 0.005 × 10^9^/L corresponding to one half of the minimum value of the measurements. For the multivariable analysis, all potential co-variables were included in the model and analyzed with backwards elimination. The final model included recipient age, stem cell source, ATG, and the IL-7Rα genotype. In an additional backwards elimination model, aGVHD and cGVHD were included as time-dependent variables and remained significant together with recipient age, stem cell source, ATG, and the IL-7Rα genotypes.

To confirm these results regarding T cell counts in a model including patients, who experienced death, relapse, retransplantation, or donor lymphocyte infusion within the first 360 days, we performed a pattern mixture model including all patients and with truncation at time of the event. This model analyzed the cell counts in each stratum (defined by time of event/truncation) in turn using a standard linear mixed model. The mixed model contained a random intercept by patient and, whenever feasible due to enough data in a stratum, a random slope by time since transplantation. The IL-7Rα genotypes were compared as major allele homozygotes (CC) versus heterozygotes and minor allele homozygotes combined (CT/TT) due to limited data within each stratum in the minor allele homozygous patients. Missing and truncated were similarly distributed in the genotype groups.

A two-sided *P*-value <0.05 was considered statistically significant. All statistical analyzes were performed using R statistical software version 3.2.3 (R Foundation for Statistical Computing, Vienna, Austria).

## Results

### IL-7Rα Genotypes and Transplantation Characteristics

The frequencies of the *rs6897932* genotype in donors [CC = 252 (55.8%), CT = 178 (38.7%), and TT = 30 (6.5%)] corresponded to previously reported gene frequencies, and the distribution of genotypes met the criteria for Hardy–Weinberg equilibrium.

Table 1 shows the transplantation characteristics divided among the *rs6897932* genotypes. No significant differences were found between patients in the three different groups.

**Table 1:** Transplantation characteristics by donor Interleukin-7 receptor α-chain genotype *rs6897932*.

Characteristics	CC (*n* = 252)	CT (*n* = 178)	TT (*n* = 30)	*P*-value
**Age at transplantation (years), median (range)**				
Recipients	25.4 (0.4–63.0)	27.8 (0.3–60.0)	27.6 (0.9–50.6)	0.61
Donors	34.0 (2.8–71.6)	33.0 (1.1–59.5)	35.6 (16.1–60.8)	0.27
**Disease at transplantation, no. of patients (%)**				
Acute myeoloid leukemia	78 (31%)	50 (28%)	8 (27%)	0.89
Acute lymphoblastic leukemia	62 (25%)	48 (27%)	8 (27%)	
Other malignancies	65 (26%)	43 (24%)	10 (33%)	
Benign disorders	47 (19%)	37 (21%)	4 (13%)	
**Donor type, no. (%)**				
HLA-identical. siblings	78 (31%)	57 (32%)	12 (40%)	0.81
HLA-matched unrelated donors	133 (53%)	97 (54%)	14 (47%)	
HLA-mismatched unrelated donors	41 (16%)	24 (13%)	4 (13%)	
**Stem cell source, no. (%)**				
Bone marrow stem cells	181 (72%)	130 (73%)	18 (60%)	0.34
Peripheral blood stem cells, G-CSF mobilized	71 (28%)	48 (27%)	12 (40%)	
**Total nucleated cell dose infused ×10^6^/kg recipient wt, median**	4	3.6	5	0.29
**Conditioning regimen, no. of patients (%)**				
TBI + CY or Etoposide	156 (62%)	116 (65%)	21 (70%)	0.61
Chemotherapy alone	96 (38%)	62 (35%)	9 (30%)	
**Anti-thymocyte globulin as part of conditioning regimen, no. (%)**				
Yes	105 (42%)	65 (37%)	9 (30%)	0.34
No	147 (58%)	113 (63%)	21 (70%)	
**Sex-mismatch (female donor to male recipient), no. (%)**				
Yes	37 (15%)	27 (15%)	7 (23%)	0.46
No	215 (85%)	151 (85%)	23 (77%)	
**CMV IgG mismatch, no. (%)**				
Yes	91 (36%)	61 (34%)	10 (33%)	0.86
No	161 (64%)	117 (66%)	20 (67%)	

### Donor *rs6897932* Genotype and aGVHD

**Figure 1:**
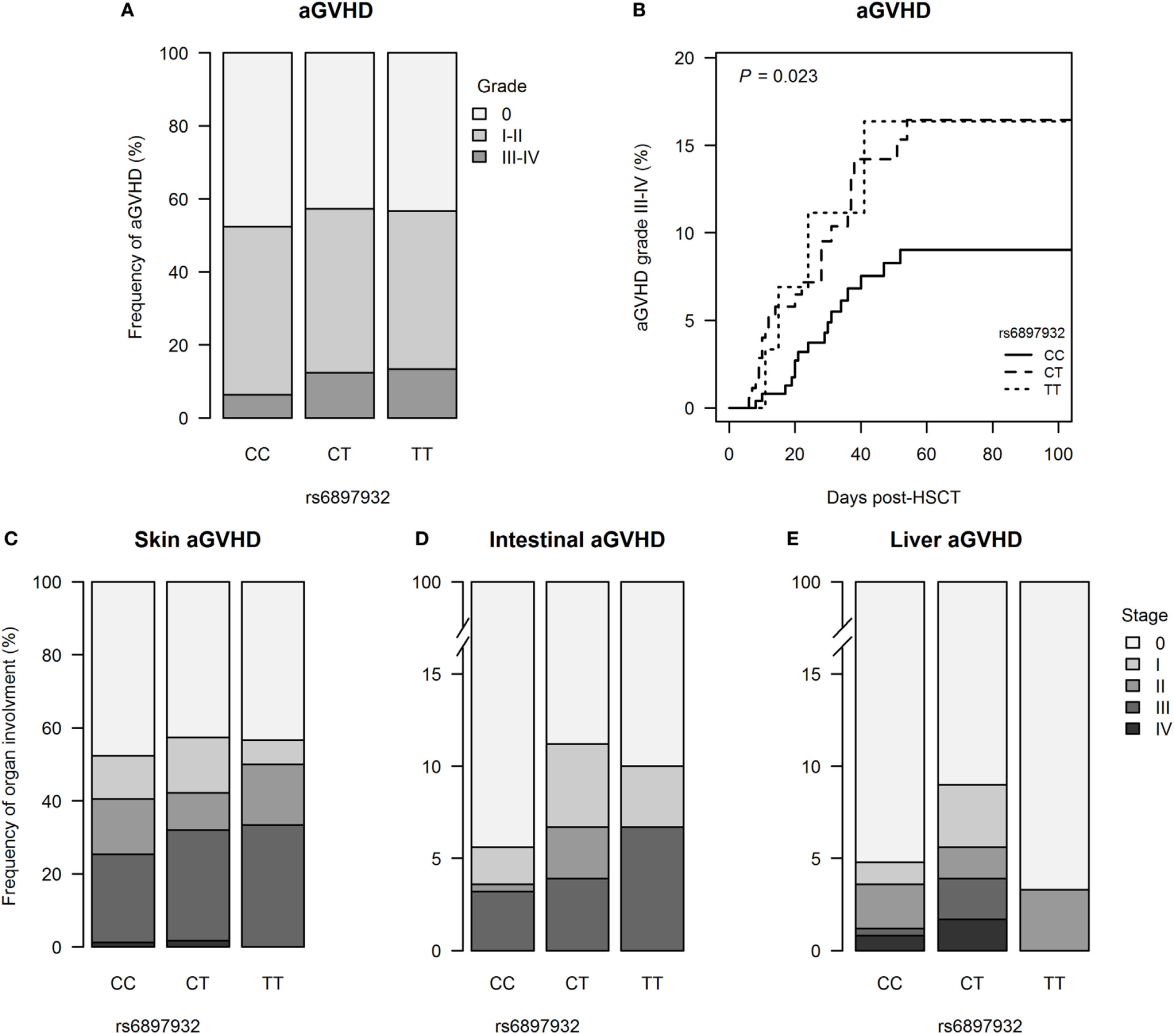
Incidence of acute graft-versus-host disease (aGVHD) following hematopoietic stem cell transplantation (HSCT) according to donor’s genotype in the Interleukin-7 receptor α-chain single nucleotide polymorphism *rs6897932*. **(A)** Relative frequency of aGVHD and **(B)** Kaplan–Meier estimates of aGVHD grades III–IV. **(C–E)** Organ staging of aGVHD. The *P*-value indicates the difference between donor genotype CT/TT and CC (genotype distribution: CC: *n* = 252, CT: *n* = 178, TT: *n* = 30).

251 patients (54.6%) developed aGVHD with onset at median day +24 (+6 to +106) post-HSCT; grades III–IV aGVHD were seen in 42 patients (9.1%). The risk of grades III–IV aGVHD was significantly increased in donors with one or two copies of the T allele in *rs6897932* (12.5% for CT/TT versus 6.3% for CC, *P* = 0.023) (Figure 1; Table S1 in Supplementary Material). This was confirmed in a multivariable Cox regression after adjustment for recipient age, donor type (matched sibling donor/matched unrelated donor/mismatched unrelated donor), stem cell source, cell dose/kilogram, TBI-based conditioning, ATG, and sex-mismatch (HR = 2.0, 95% CI = 1.1–3.8, *P* = 0.036 for TT/CT compared with the CC genotype).

### Donor *rs6897932* Genotype and cGVHD

**Figure 2:**
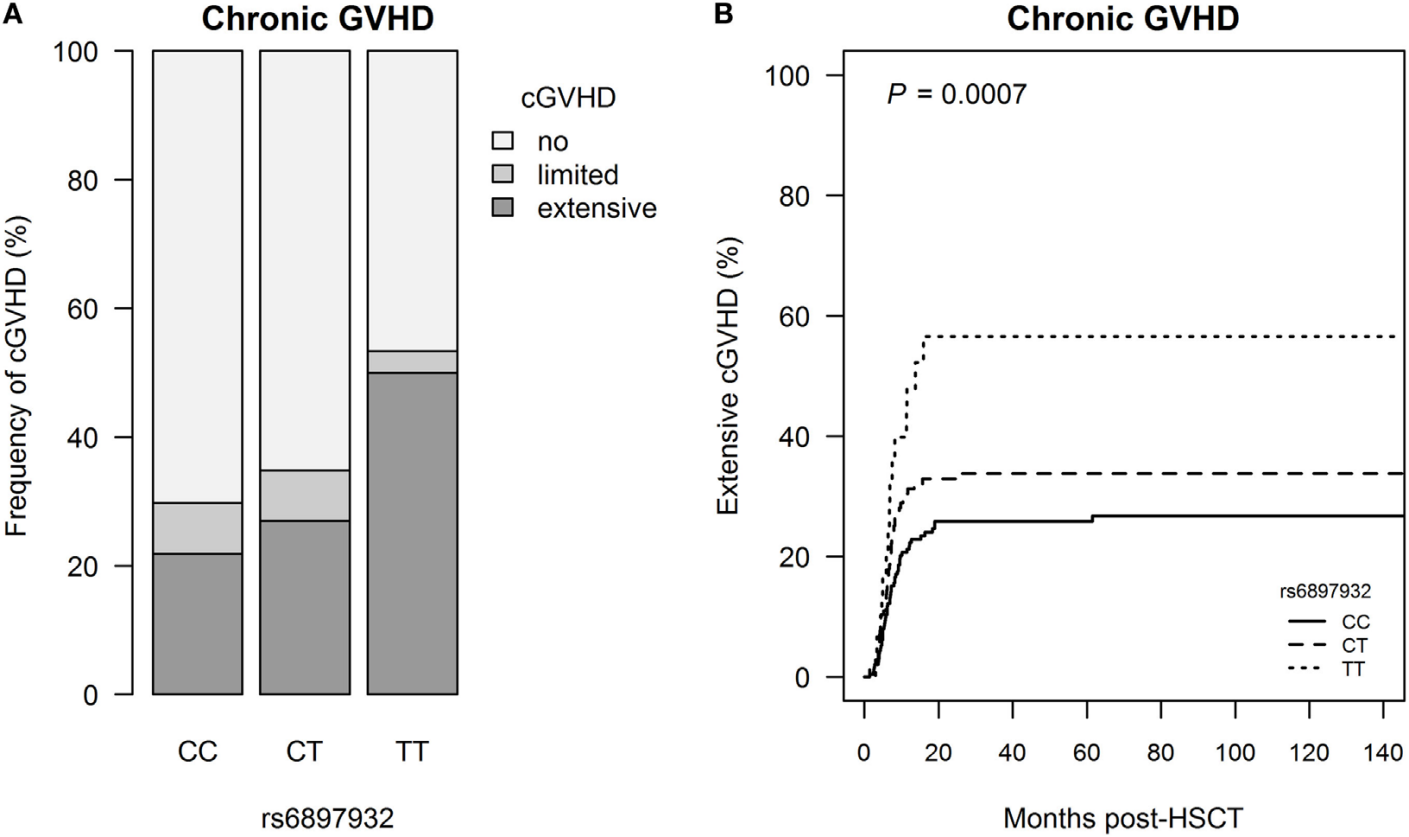
Incidence of chronic graft-versus-host disease (cGVHD) following hematopoietic stem cell transplantation (HSCT) according to donor’s genotype in the Interleukin-7 receptor α-chain single nucleotide polymorphism *rs6897932*. **(A)** Relative frequency of cGVHD and **(B)** Kaplan–Meier estimates of extensive cGVHD. The *P*-value indicates the difference between donor genotype TT and CC (genotype distribution: CC: *n* = 252, CT: *n* = 178, TT: *n* = 30).

Extensive cGVHD occurred in 118 patients (25.7%) at median day +201 (+42 to +2,088), and this was significantly associated with donor *rs6897932* genotype with a step-wise increased risk of cGVHD for each T allele (CC: 21.8%, CT: 27.0%, and TT: 50.0%, *P* = 0.0031) (Figure 2; Table S1 in Supplementary Material). The risk of extensive cGVHD was significantly increased for the TT versus CC genotype in a multivariable Cox regression adjusting for recipient age, donor type (matched sibling donor/matched unrelated donor/mismatched unrelated donor), stem cell source, cell dose/kilogram, TBI-based conditioning, ATG, and sex-mismatch (HR = 2.0, 95% CI = 1.1–3.6, *P* = 0.025).

To address if the effect of the donor genotype on chronic GVHD was mediated by the increased risk of aGVHD, we included aGVHD in a Cox regression model also adjusting for recipient age, donor type, stem cell source, cell dose/kilogram, TBI-based conditioning, ATG, and sex-mismatch. In this modified model, the donor *rs6897932* genotype remained an independent risk factor of extensive cGVHD (HR = 1.9, 95% CI = 1.0–3.5, *P* = 0.035).

### Donor *rs6897932* Genotype and Viral Infections

123 patients (26.7%) developed therapy-requiring CMV infection at median day +49 post-HSCT, and 34 patients (7.4%) developed therapy-requiring EBV infection at median day +57.

**Figure 3:**
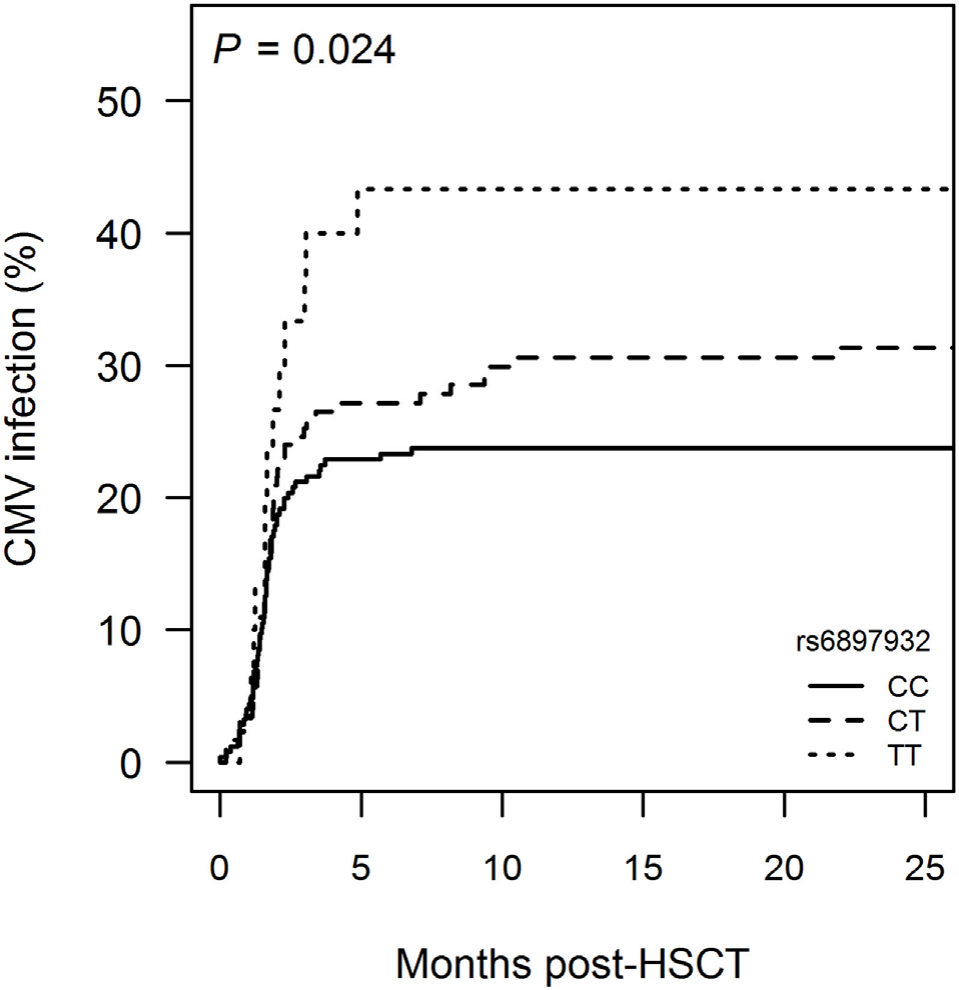
Incidence of cytomegalovirus (CMV) infection following hematopoietic stem cell transplantation (HSCT) according to donor’s genotype in the Interleukin-7 receptor α-chain single nucleotide polymorphism *rs6897932*. Kaplan–Meier estimates of treatment-requiring CMV infection are shown. The *P*-value indicates the difference between donor genotype TT and CC (genotype distribution: CC: *n* = 252, CT: *n* = 178, TT: *n* = 30).

Donor *rs6897932* TT genotype was associated with a higher occurrence of CMV infection compared with CC (CC: 23.8%, CT: 31.4%, and TT: 43.3%, *P* = 0.053) (Figure 3; Table S1 in Supplementary Material). The risk of CMV infection post-HSCT was significantly increased with HR = 2.3 (95% CI = 1.2–4.3, *P* = 0.0083) for TT versus CC genotype in a multivariable Cox regression model adjusting for recipient age, donor type (matched sibling donor/matched unrelated donor/mismatched unrelated donor), stem cell source, cell dose, TBI-based conditioning, ATG and CMV mismatch between donor and recipient (D−R+ or D+R−). No association with EBV infection was observed.

### Donor *rs6897932* Genotype and Early Lymphocyte Reconstitution

Lymphocyte recovery with >10^9^ lymphocytes/L occurred in 427 patients (92.8%) within the first year post-HSCT. There was no association between donor *rs6897932* genotype and time to lymphocyte recovery.

We further investigated the impact *rs6897932* on recovery of T and B lymphocyte subsets within 1 year post-HSCT. Patients were excluded from the analysis in case of death, relapse, retransplantation, or donor lymphocyte infusion from the date of the event.

**Figure 4:**
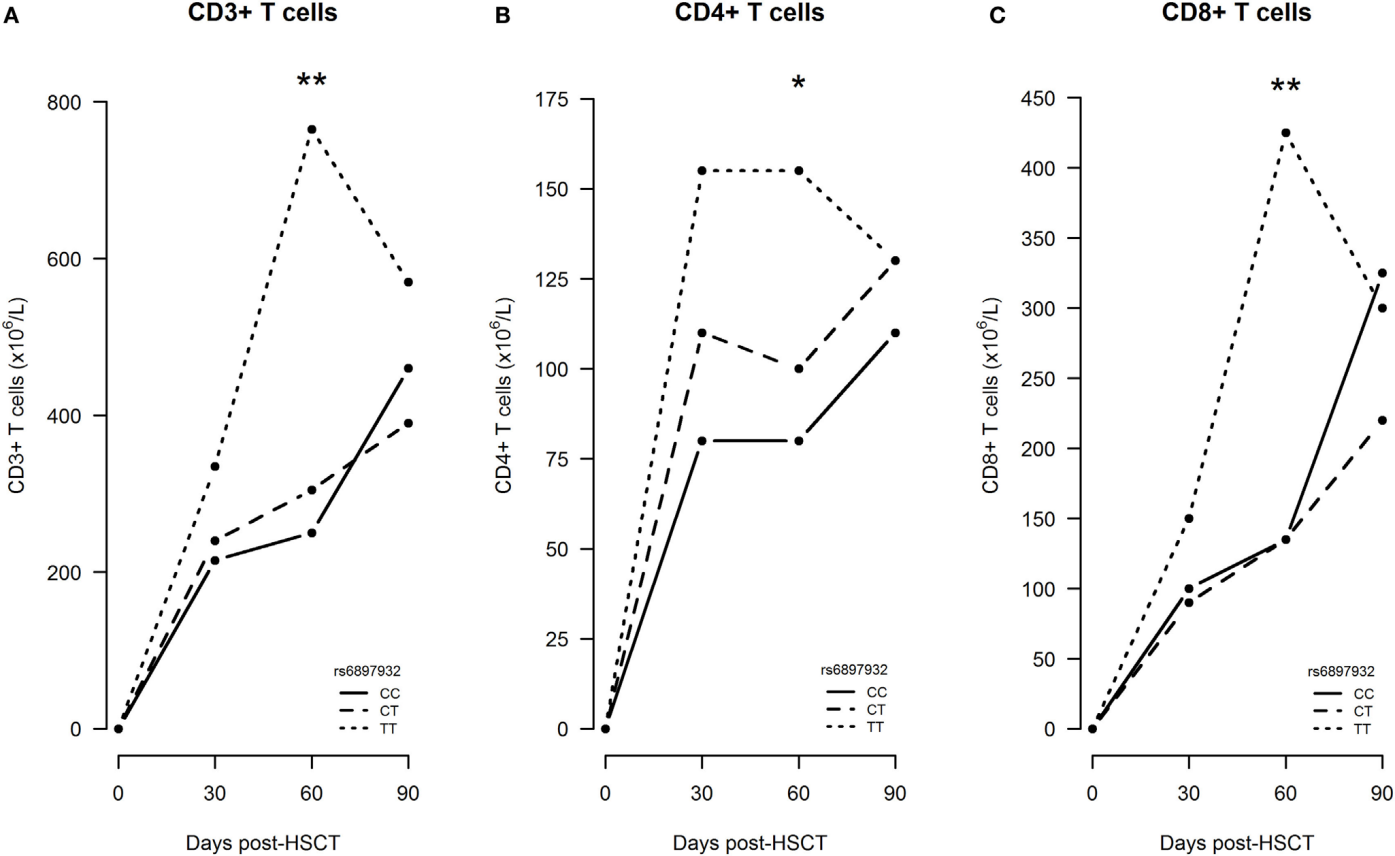
Early immune reconstitution following hematopoietic stem cell transplantation (HSCT) according to donor’s genotype in the Interleukin-7 receptor α-chain single nucleotide polymorphism *rs6897932*. **(A–C)** Median CD3+, CD4+, and CD8+ T cell counts from time of transplantation to 3 months post-HSCT. The *P*-values indicate the difference between donor genotype TT and CC at the specific time point in a longitudinal linear mixed model (*P < 0.05, **P < 0.01) (genotype distribution: CC: *n* = 108, CT: *n* = 86, TT: *n* = 18).

First, we studied the impact on immune reconstitution in a linear mixed model only including patients, who did not experience an event within the first 360 days (*n* = 212). In an univariable model, the donor *rs6897932* genotype TT was associated with significantly increased CD3+, CD4+, and CD8+ T cells at day +60 compared with the CC genotype (Figure 4). This was confirmed in the multivariable model, where the donor TT genotype was associated with an increased number of T cell subsets (CD3+: 109% increase, *P* = 0.0096; CD4+: 64% increase, *P* = 0.038; CD8+: 133% increase, *P* = 0.011) at day +60 compared with the CC genotype, after adjustment for age, stem cell source, and ATG (Table 2). These results were similar, when also adjusting for the immunosuppressive effect of aGVHD and cGVHD by including them as time-dependent co-variables in this model (*P* = 0.0079, *P* = 0.030, and *P* = 0.0096, respectively).

**Table 2:** Longitudinal T cell reconstitution by donor Interleukin-7 receptor α-chain genotype.

	Univariable model [estimate (95% CI)]					Multivariable model [estimate (95% CI)]				

	CC	CT	*P*-value (CT versus CC)	TT	*P*-value (TT versus CC)	CC	CT	*P*-value (CT versus CC)	TT	*P*-value (TT versus CC)
**Day +30**										
CD3+	1.0	0.90 (0.61–1.32)	0.59	1.37 (0.71–2.64)	0.34	1.0	0.94 (0.67–1.33)	0.75	1.04 (0.57–1.90)	0.89
CD4+	1.0	0.97 (0.69–1.37)	0.85	1.68 (0.93–3.05)	0.088	1.0	1.03 (0.77–1.38)	0.86	1.27 (0.76–2.10)	0.36
CD8+	1.0	0.91 (0.61–1.38)	0.67	1.19 (0.58–2.44)	0.63	1.0	0.95 (0.64–1.41)	0.79	0.96 (0.48–1.92)	0.92
**Day +60**										
CD3+	1.0	1.16 (0.81–1.67)	0.42	**2.37 (1.29–4.35)**	**0.006**	1.0	1.20 (0.86–1.67)	0.30	**2.09 (1.20–3.66)**	**0.0097**
CD4+	1.0	1.08 (0.78–1.50)	0.63	**1.94 (1.13–3.37)**	**0.017**	1.0	1.13 (0.86–1.50)	0.38	**1.64 (1.03–2.61)**	**0.038**
CD8+	1.0	1.24 (0.83–1.86)	0.29	**2.53 (1.29–4.96)**	**0.007**	1.0	1.26 (0.85–1.86)	0.24	**2.33 (1.21–4.47)**	**0.011**
**Day +90**										
CD3+	1.0	1.02 (0.73–1.44)	0.89	1.36 (0.70–2.64)	0.36	1.0	1.02 (0.74–1.39)	0.91	1.22 (0.66–2.27)	0.53
CD4+	1.0	0.99 (0.74–1.34)	0.97	1.53 (0.85–2.73)	0.16	1.0	1.01 (0.78–1.30)	0.96	1.28 (0.77–2.15)	0.34
CD8+	1.0	1.03 (0.71–1.51)	0.86	1.35 (0.64–2.83)	0.43	1.0	1.03 (0.71–1.48)	0.89	1.26 (0.61–2.59)	0.53
**Day +180**										
CD3+	1.0	1.00 (0.74–1.36)	0.99	0.94 (0.55–1.59)	0.81	1.0	0.99 (0.74–1.33)	0.95	0.86 (0.52–1.43)	0.56
CD4+	1.0	0.98 (0.75–1.28)	0.86	1.13 (0.71–1.80)	0.61	1.0	0.97 (0.76–1.23)	0.80	1.01 (0.66–1.53)	0.97
CD8+	1.0	0.94 (0.66–1.35)	0.74	0.80 (0.43–1.49)	0.48	1.0	0.93 (0.66–1.33)	0.71	0.76 (0.41–1.40)	0.38
**Day +360**										
CD3+	1.0	0.97 (0.72–1.31)	0.83	0.93 (0.53–1.63)	0.79	1.0	0.94 (0.71–1.24)	0.67	0.91 (0.54–1.55)	0.74
CD4+	1.0	0.92 (0.67–1.25)	0.59	1.04 (0.58–1.86)	0.90	1.0	0.89 (0.68–1.16)	0.38	0.98 (0.59–1.63)	0.95
CD8+	1.0	0.98 (0.69–1.39)	0.90	0.87 (0.45–1.66)	0.67	1.0	0.95 (0.68–1.34)	0.77	0.86 (0.45–1.63)	0.64

*Significant associations are written in bold*.

Next, we investigated the significance of IL-7Rα donor genotypes in a pattern mixture model with truncation due to death, relapse, retransplantation, or donor lymphocyte infusion to confirm the first results in a cohort including patients who experienced an event (*n* = 268). At each time of follow-up, the expected change in median cell count were estimated and compared between the genotype groups. The two groups CT and TT were merged to be able to estimate all parameters, due to the limited number of donors with the TT genotype. In this model, no significant difference between cell counts for the *rs6897932* genotype was found at any time point, most likely due to the limited data in each strata. Notably, missing and truncated patients were similarly distributed in the two genotype-defined groups.

### Donor *rs6897932* Genotype and Late Immunity

**Figure 5:**
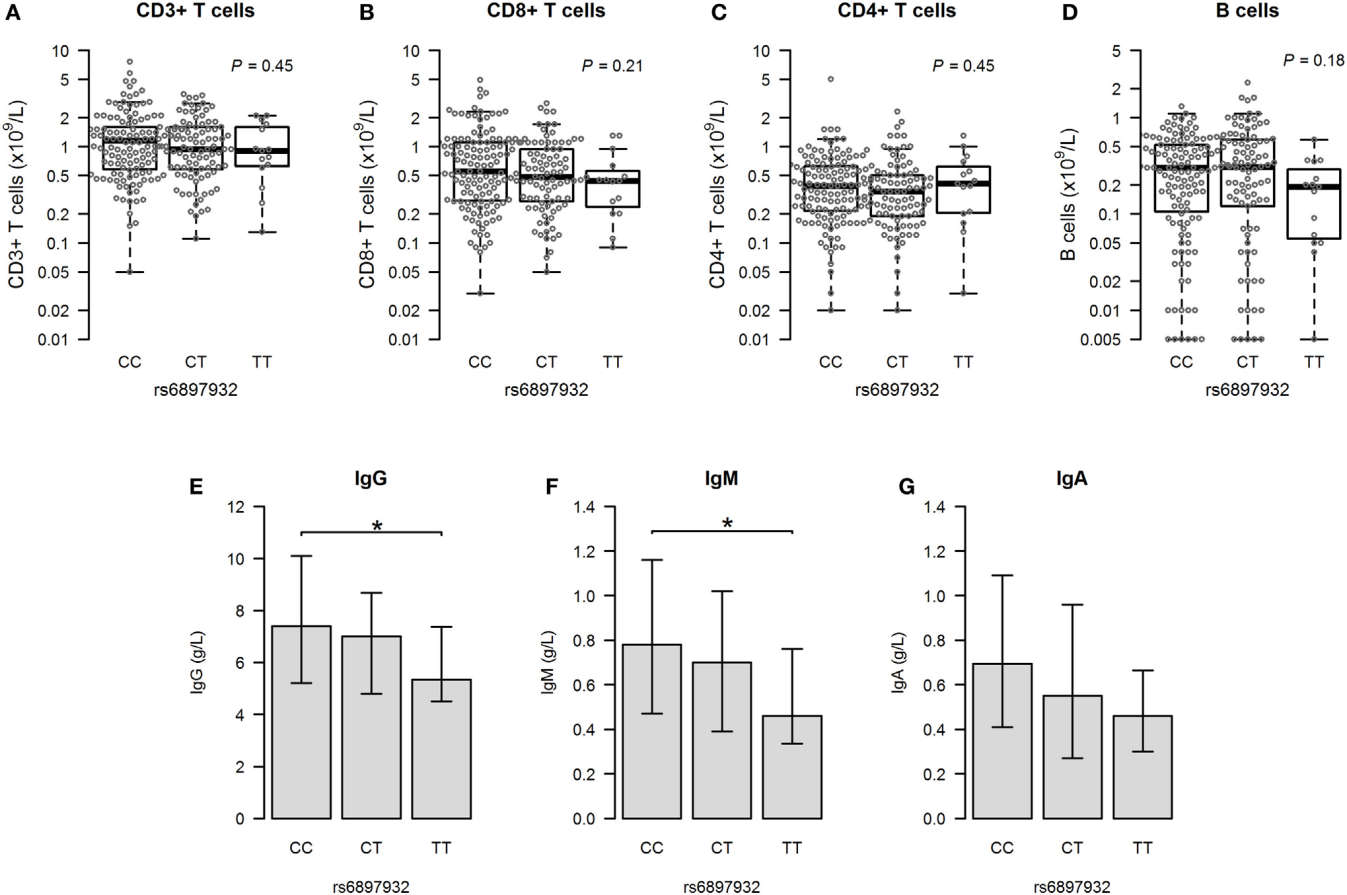
Immunity at 1 year post-hematopoietic stem cell transplantation according to donor’s genotype in the Interleukin-7 receptor α-chain single nucleotide polymorphism *rs6897932*. **(A–D)** Total counts of CD3+, CD8+, and CD4+ T cells and CD19+ B cells. **(E–G)** Plasma levels of immunoglobulins (median plus quartiles). The *P*-values indicate the difference between donor genotype TT and CC (*P < 0.05) (genotype distribution: CC: *n* = 128, CT: *n* = 90, TT: *n* = 15).

We next assessed the major immune parameters 1 year after HSCT. Measurements of T and B cells were available in 233 (72.6%) patients having no events before this time point. There was no difference in cell counts of any lymphocyte subsets according to the *rs6897932* genotype in a linear regression model both before and after adjustment for age, stem cell source, and ATG (Figures 5A–D; Table 2).

We looked into the level of immunoglobulins to evaluate the functional interaction between T and B cells. The donor *rs6897932* TT genotype was associated with significantly lower levels of IgG and IgM compared to the CC genotype 1 year post-HSCT (Figures 5E–G), although no difference was observed before transplantation. This finding remained significant for IgG in a multivariable model adjusting for age, stem cell source, and ATG (*P* = 0.027). However, this decrease in immunoglobulin levels was also strongly associated with occurrence of aGVHD and cGVHD, suggesting that the immunosuppressive effect of *rs6897932* on late immunity might be mediated through an increased allo-response.

### Donor *rs6897932* Genotype and Mortality

We next investigated whether the *rs6897932* donor genotype also influenced post-transplant mortality. 156 patients (33.9%) died within the follow-up time of 6.9 years (range: 1.9–12.8). Of patients transplanted for a malignant disease, 78 patients (21.0%) died of TRM and 81 patients (21.8%) relapsed.

**Figure 6:**
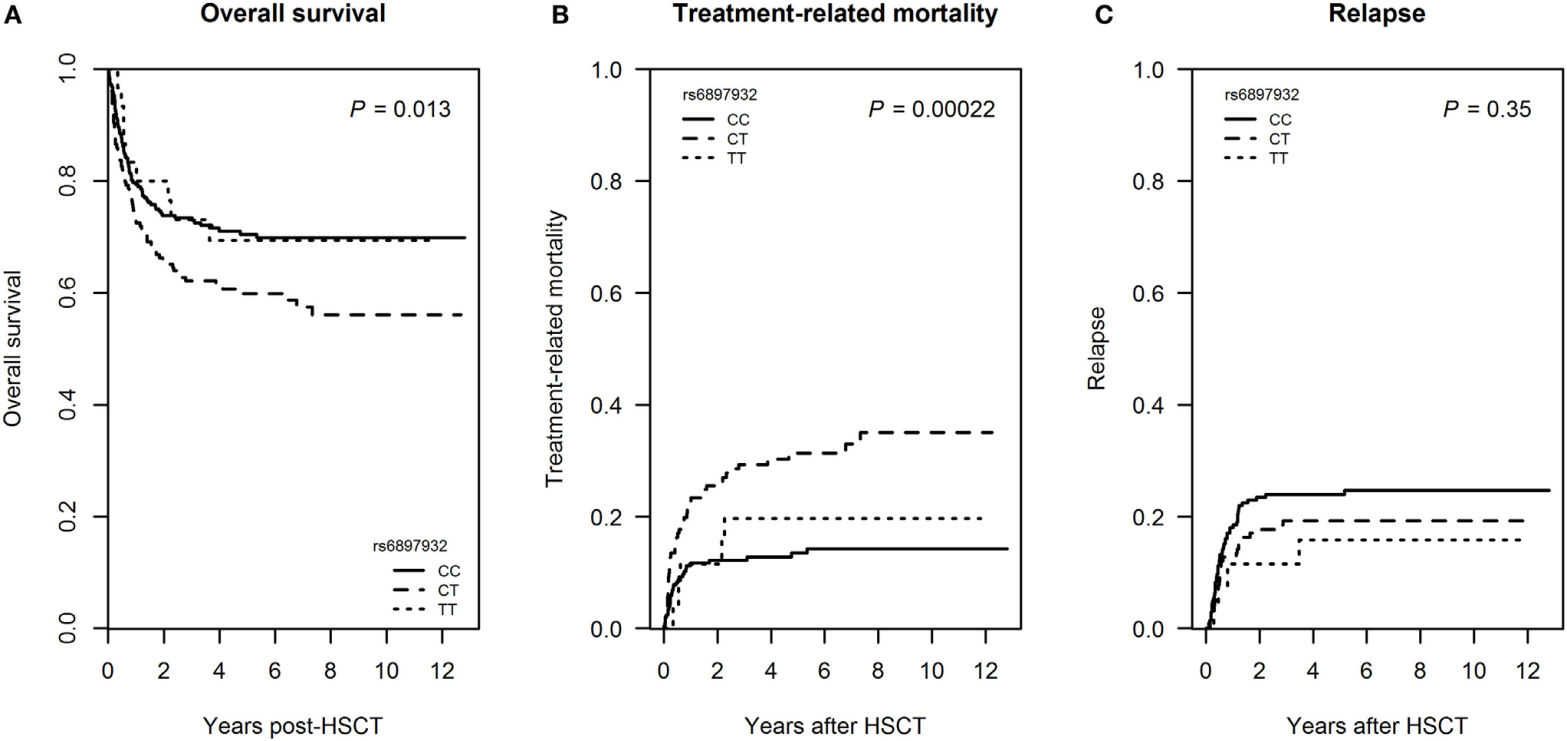
Mortality following hematopoietic stem cell transplantation (HSCT) according to donor’s genotype in the Interleukin-7 receptor α-chain single nucleotide polymorphism *rs6897932*. **(A)** Overall survival, **(B)** treatment-related mortality (TRM), and **(C)** relapse frequency. Overall survival is estimated by Kaplan–Meier, while TRM and relapse are estimated by a Fine–Gray competing risk model. The *P*-values indicate the difference between donor genotype CT and CC (genotype distribution: CC: *n* = 252, CT: *n* = 178, TT: *n* = 30).

Donor carriage of the *rs6897932* CT genotype was associated with inferior overall survival in an univariable Cox regression model (HR = 1.50, *P* = 0.013) (Figure 6A; Table S1 in Supplementary Material) as well as in a multivariable model adjusting for recipient age, diagnosis, donor type, stem cell source, TBI-based conditioning, and ATG (HR = 1.7 for CT versus CC genotype, 95% CI = 1.2–2.3, *P* = 0.0027).

In a competing risk model, donor *rs6897932* CT genotype was associated with increased TRM in a univariable model (cumulative incidence estimates: CC = 14.3%, CT = 35.0, TT = 19.6%, *P* = 0.00022 for CT versus CC genotype), but not with the risk of relapse (*P* = 0.35) (Figures 6B,C; Table S1 in Supplementary Material). The significant association with TRM was confirmed in a multivariable competing risk model adjusted for recipient age, donor type, stem cell source, TBI-based conditioning, and ATG (HR = 2.3, 95% CI = 1.3–4.0, *P* = 0.0047 for CT versus CC). We were not able to demonstrate a significant effect of TT homozygosity on TRM most likely reflecting the low prevalence of this genotype.

## Discussion

Despite the marked improvements during the recent years, allogeneic HSCT is still challenged by severe treatment-related complications (27). Both aGVHD and cGVHD cause significant morbidity and mortality after HSCT, and the long-term immunosuppressive treatment of these complications may have limited efficacy although still hampering the immune responses toward infections and the remaining leukemic cells (28). Thus, identification of risk factors for development of alloreactivity and immune dysfunction may be an important step toward a more effective risk stratification to prevent these complications.

Our data show that donor *rs6897932* TT genotype in the IL-7Rα is associated with increased risk of both aGVHD and cGVHD, CMV infection, and faster reconstitution of T cells. These results indicate that genotyping of *rs6897932* could help to individualize conditioning regimens and GVHD prophylaxis, or potentially be included as a supplementary criteria for donor selection along with HLA-typing. As alloreactivity and the graft-versus-leukemia effect are often closely associated (27), it is of particular interest that the impact of the *rs6897932* genotype is restricted to treatment-related complications with no significant impact on relapse.

The *rs6897932* SNP has been studied previously in low-powered or heterogeneous multicenter studies with conflicting results concerning aGVHD and mortality (21–23, 29, 30). A major strength of the present study is the large number of HSCT patients studied within a single institution with an ethnically homogeneous population and a uniform registration of complications. However, in comparison with genetic studies linking candidate SNPs to development of disease in general, our cohort is relatively small and results should be taken with caution, especially considering the low frequency of the risk genotype.

Interleukin-7 is a cytokine with effects on both peripheral expansion of T cells and thymic T cell production that is known to decline with age (31, 32). Accordingly, we found it important to address age-related differences in the impact of the IL-7Rα SNP. In an age-stratified analysis, we found similar associations with clinical outcomes and T cell reconstitution in adult and pediatric patients suggesting that the effects of the genotype were independent on age-related changes in thymic function. In line with this, the IL-7Rα SNP appeared to affect T cell numbers at an early stage before day 100 where thymic output in the form of T cell receptor excision circles cannot be detected (33–35), suggesting that *rs6897932* is mainly affecting the peripheral expansion of T cells of importance for both children and adults in the early post-transplant period.

The biological background for the impact of *rs6897932* on immune dysregulation in allo- and autoimmunity has been addressed previously. Studies in conditions with elevated IL-7 levels, due to lymphopenia or pharmacologic administration of IL-7, indicate that IL-7 in high concentrations may enhance the proliferative responses even to weak *self*-antigens (36–38). Therefore, it is likely that IL-7 may also drive peripheral expansion of naïve alloreactive T cell clones early after HSCT, where IL-7 levels are highly increased (39–41). Furthermore, recent studies suggest that IL-7 specifically enhances the proliferation of pro-inflammatory T cell subsets (42, 43) and reduce the functional capacity of regulatory T cells to suppress proliferation and cytokine production (44). In line with this, elevated IL-7 levels are associated with development of aGVHD after myeloablative HSCT and is increased in autoimmune diseases (39–41, 45).

The mechanism by which *rs6897932* impacts outcome of HSCT is most likely related to an altered degree of binding of IL-7 to soluble IL-7R (sIL-7R). The T allele in *rs6897932* causes an amino-acid substitution in the transmembrane region of the IL-7Rα gene which reduces alternative splicing of this domain. This process results in increased expression of membrane-bound IL-7R and decreased generation of sIL-7R (46, 47). Since sIL-7R acts as an inhibitor of IL-7 signaling *in vitro* (48), *rs6897932* may affect IL-7 activity not only by diminishing sIL-7R levels, but also through increased expression of membrane-bound IL-7Rα.

Several lines of evidence suggest that the T allele of *rs6897932* increases IL-7 activity. First, the T allele is associated with faster CD3+, CD4+, and CD8+ T cell reconstitution early post-HSCT as shown here. These results correspond to studies in T cell depletion caused by human immunodeficiency virus, where the *rs6897932* T allele and low-sIL-7R levels were associated with a more rapid CD4+ T cell recovery after anti-retroviral treatment (49). Furthermore, *rs6897932* T allele has been associated with increased intracellular STAT-5 signaling in CD4+ T cells after *in vitro* stimulation with IL-7, resulting in increased cellular proliferation (50). In allogeneic HSCT, the increased homeostatic proliferation and survival of T cells from donors with the *rs6897932* TT genotype are likely a contributing reason for the increased risk of GVHD observed here.

Secondly, the T allele is associated with reduced plasma levels of sIL-7R that are related to aGVHD (30, 51). This is, however, in contrast to the findings in chronic inflammatory diseases, where the *rs6897932* C allele and elevated sIL-7R levels have been identified as risk factors for developing multiple sclerosis, ulcerative colitis, sarcoidosis, inhalation allergy, and type 1 diabetes (17–20, 52–57). The explanation for this apparent inconsistency may relate to the limited levels of IL-7 seen in these autoimmune conditions compared with the HSCT setting, where IL-7 levels are many-fold above normal levels. A recent study suggested that sIL-7R is inhibitory at short-term, while overall potentiating the bioavailability of IL-7 by protecting the cytokine from consumption (58). In HSCT, however, the scenario is much different due to the supra-physiological levels of IL-7 in combination with severely depressed sIL-7R levels in the early post-transplant period (30, 51). Under these extreme conditions, even a short-term inhibition mediated by high sIL-7R may be enough to protect against alloreactivity, and thereby explain the lower incidence of GVHD in transplantations with a donor CC genotype.

Recent studies also suggest an importance of IL-7Rα mediated signals for normal human B cell production (59), although patients with SCID caused by a defect IL-7Rα signaling have normal B cell numbers (14). Our results do not suggest an association between IL-7Rα SNPs and quantitative B cell reconstitution following HSCT, but show significantly decreased IgG levels after 1 year in patients transplanted with the *rs6897932* TT genotype. This could not be related to a difference in T or B cell counts that were similar among the genotypes at this time point, although a functional impact on T cell subsets required for isotype switch and antibody production cannot be excluded. Importantly, however, our multivariable analysis suggested that the effect of this genotype on immunoglobulin levels may be mediated through cGVHD, suppressing bone marrow function (including the plasma cells) and thymopoiesis directly as well as indirectly by its immunosuppressive treatment (60). A potential confounding factor for these findings could be the administration of Rituximab for EBV infection, although this is most likely a minor factor in our study due the low numbers of patients receiving treatment and the equal distribution of EBV infection among the different *rs6897932* genotypes.

In conclusion, we have presented solid evidence that the donor *rs6897932* genotype of the IL-7Rα predicts aGVHD and cGVHD, CMV infection and TRM following allogeneic HSCT, without altering the risk of relapse. The biological background for the impact of this SNP may be alterations in the levels of soluble and membrane-bound IL-7R leading to stronger IL-7 signaling. Prospective studies should address the role of IL-7R genotyping as part of the donor selection process to reduce the incidence of treatment-related complications and its application as a pre-transplant biomarker identifying patients at risk and guiding prophylactic treatment.

## Ethics Statement

This study was carried out in accordance with the recommendations of the ethics committee of the Capital Region of Denmark. All subjects and/or their legal guardians gave written informed consent in accordance with the Declaration of Helsinki.

## Author Contributions

KK, AP, LR, and KM conceived and designed the study. KK, CH, HS, and KM collected the donor samples and the clinical data. CE performed the genotyping. KK and KM analyzed the data and drafted the manuscript. All authors revised the manuscript.

## Conflict of Interest Statement

AP is affiliated with Merck A/S; however, the present work is unrelated to this affiliation. All other authors report no conflicts of interest. The authors alone are responsible for the content and writing of the paper.

## Abbreviations

HSCT, hematopoietic stem cell transplantation; GVHD, graft-versus-host disease; IL-7Rα, interleukin-7 receptor α-chain; TBI, total body irradiation; ATG, anti-thymocyte globulin; CMV, cytomegalovirus; EBV, Epstein–Barr virus.

## References

[1] Storek J Gooley T Witherspoon RP Sullivan KM Storb R. Infectious morbidity in long-term survivors of allogeneic marrow transplantation is associated with low CD4 T cell counts. Am J Hematol (1997) 54:131–8. 10.1002/(SICI)1096-8652(199702)54:2<131::AID-AJH6>3.0.CO;2-Y 9034287

[2] Kim DH Sohn SK Won DI Lee NY Suh JS Lee KB Rapid helper T-cell recovery above 200 x 10^6^/l at 3 months correlates to successful transplant outcomes after allogeneic stem cell transplantation. Bone Marrow Transplant (2006) 37:1119–28. 10.1038/sj.bmt.1705381 16699530

[3] Ferrara JLM Levine JE Reddy P Holler E. Graft-versus-host disease. Lancet (2009) 373:1550–61. 10.1016/S0140-6736(09)60237-3 19282026 PMC2735047

[4] Plum J De Smedt M Leclercq G Verhasselt B Vandekerckhove B. Interleukin-7 is a critical growth factor in early human T-cell development. Blood (1996) 88:4239–45. 8943859

[5] Fry TJ Mackall CL. The many faces of IL-7: from lymphopoiesis to peripheral T cell maintenance. J Immunol (2005) 174:6571–6. 10.4049/jimmunol.174.11.6571 15905493

[6] Carrette F Surh CD. IL-7 signaling and CD127 receptor regulation in the control of T cell homeostasis. Semin Immunol (2012) 24:209–17. 10.1016/j.smim.2012.04.010 22551764 PMC3367861

[7] Jiang Q Li WQ Aiello FB Mazzucchelli R Asefa B Khaled AR Cell biology of IL-7, a key lymphotrophin. Cytokine Growth Factor Rev (2005) 16:513–33. 10.1016/j.cytogfr.2005.05.004 15996891

[8] Reche PA Soumelis V Gorman DM Clifford T Liu M Travis M Human thymic stromal lymphopoietin preferentially stimulates myeloid cells. J Immunol (2001) 167:336–43. 10.4049/jimmunol.167.1.336 11418668

[9] Ziegler SF Artis D. Sensing the outside world: TSLP regulates barrier immunity. Nat Immunol (2010) 11:289–93. 10.1038/ni.1852 20300138 PMC2924817

[10] Al-Shami A Spolski R Kelly J Fry T Schwartzberg PL Pandey A A role for thymic stromal lymphopoietin in CD4(+) T cell development. J Exp Med (2004) 200:159–68. 10.1084/jem.20031975 15263024 PMC2212020

[11] Lei L Zhang Y Yao W Kaplan MH Zhou B. Thymic stromal lymphopoietin interferes with airway tolerance by suppressing the generation of antigen-specific regulatory T cells. J Immunol (2011) 186:2254–61. 10.4049/jimmunol.1002503 21242516 PMC3125594

[12] Moret FM Hack CE Van Der Wurff-Jacobs KMG Radstake TRDJ Lafeber FPJG van Roon JAG. Thymic stromal lymphopoietin, a novel proinflammatory mediator in rheumatoid arthritis that potently activates CD1c+ myeloid dendritic cells to attract and stimulate T cells. Arthritis Rheumatol (2014) 66:1176–84. 10.1002/art.38338 24782181

[13] Mazzucchelli R Durum SK. Interleukin-7 receptor expression: intelligent design. Nat Rev Immunol (2007) 7:144–54. 10.1038/nri2023 17259970

[14] Puel A Ziegler SF Buckley RH Leonard WJ. Defective IL7R expression in T(-)B(+)NK(+) severe combined immunodeficiency. Nat Genet (1998) 20:394–7. 10.1038/3877 9843216

[15] Zenatti PP Ribeiro D Li W Zuurbier L Silva MC Paganin M Oncogenic IL7R gain-of-function mutations in childhood T-cell acute lymphoblastic leukemia. Nat Genet (2011) 43:932–9. 10.1038/ng.924 21892159 PMC7424552

[16] Silva A Laranjeira ABA Martins LR Cardoso BA Demengeot J Yunes JA IL-7 contributes to the progression of human T-cell acute lymphoblastic leukemias. Cancer Res (2011) 71:4780–9. 10.1158/0008-5472.CAN-10-3606 21593192

[17] Teutsch S Booth D Identification of 11 novel and common single nucleotide polymorphisms in the interleukin-7 receptor-α gene and their associations with multiple sclerosis. Eur J Hum Genet (2003) 11:509–15. 10.1038/sj.ejhg.5200994 12825072

[18] Lundmark F Duvefelt K Iacobaeus E Kockum I Wallström E Khademi M Variation in interleukin 7 receptor alpha chain (IL7R) influences risk of multiple sclerosis. Nat Genet (2007) 39:1108–13. 10.1038/ng2106 17660816

[19] Heron M Grutters JC van Moorsel CHM Ruven HJT Huizinga TWJ van der Helm-van Mil AHM Variation in IL7R predisposes to sarcoid inflammation. Genes Immun (2009) 10:647–53. 10.1038/gene.2009.55 19626041

[20] Anderson CA Boucher G Lees CW Franke A D’Amato M Taylor KD Corrigendum: meta-analysis identifies 29 additional ulcerative colitis risk loci, increasing the number of confirmed associations to 47. Nat Genet (2011) 43:246–52. 10.1038/ng0911-919b 21297633 PMC3084597

[21] Shamim Z Ryder LP Heilmann C Madsen H Lauersen H Andersen PK Genetic polymorphisms in the genes encoding human interleukin-7 receptor-alpha: prognostic significance in allogeneic stem cell transplantation. Bone Marrow Transplant (2006) 37:485–91. 10.1038/sj.bmt.1705277 16435014

[22] Shamim Z Ryder LP Christensen IJ Toubert A Norden J Collin M Prognostic significance of interleukin-7 receptor-α gene polymorphisms in allogeneic stem-cell transplantation: a confirmatory study. Transplantation (2011) 91:731–6. 10.1097/TP.0b013e31820f08b2 21326139

[23] Shamim Z Spellman S Haagenson M Wang T Lee SJ Ryder LP Polymorphism in the interleukin-7 receptor-alpha and outcome after allogeneic hematopoietic cell transplantation with matched unrelated donor. Scand J Immunol (2013) 78:214–20. 10.1111/sji.12077 23692589 PMC3982186

[24] Gratwohl A Carreras E Principles of conditioning. In: Apperley J Carreras E Gluckman E Masszi T, editors. The EBMT Handbook. (2012).

[25] Enevold C Oturai AB Sørensen PS Ryder LP Koch-Henriksen N Bendtzen K. Multiple sclerosis and polymorphisms of innate pattern recognition receptors TLR1-10, NOD1-2, DDX58, and IFIH1. J Neuroimmunol (2009) 212:125–31. 10.1016/j.jneuroim.2009.04.008 19450885

[26] Sullivan K Mannucci A Kimpton C Gill P. A rapid and quantitative DNA sex test: fluorescence-based PCR analysis of X-Y homologous gene amelogenin. Biotechniques (1993) 15:636–41. 8251166

[27] Singh AK Mcguirk JP. Allogeneic stem cell transplantation: a historical and scientific overview. Cancer Res (2016) 76:6445–52. 10.1158/0008-5472.CAN-16-1311 27784742

[28] Im A Hakim FT Pavletic SZ Novel targets in the treatment of chronic graft-versus-host disease. Leukemia (2017) 31:543–54. 10.1038/leu.2016.367 27899803

[29] Azarpira N Dehghani M Aghdaie MH Darai M. Interleukin-7 receptor-alpha gene polymorphisms in bone marrow transplant recipients. Mol Biol Rep (2010) 37:27–31. 10.1007/s11033-009-9488-4 19253027

[30] Kielsen K Shamim Z Thiant S Faucher S Decker W Christensen IJ Soluble Interleukin-7 receptor levels and risk of acute graft-versus-disease after allogeneic haematopoietic stem cell transplantation. Clin Immunol (2017). 10.1016/j.clim.2017.08.015 28863969

[31] Mackall CL Fleisher TA Brown MR Andrich MP Chen CC Feuerstein IM Age, thymopoiesis, and CD4+ T-lymphocyte regeneration after intensive chemotherapy. N Engl J Med (1995) 332:143–9. 10.1056/NEJM199501193320303 7800006

[32] Sauce D Larsen M Fastenackels S Roux A Gorochov G Katlama C Lymphopenia-driven homeostatic regulation of naive T cells in elderly and thymectomized young adults. J Immunol (2012) 189:5541–8. 10.4049/jimmunol.1201235 23136199

[33] Krenger W Blazar BR Holländer GA. Thymic T-cell development in allogeneic stem cell transplantation. Blood (2011) 117:6768–76. 10.1182/blood-2011-02-334623 21427289 PMC3128475

[34] Toubert A Glauzy S Douay C Clave E. Thymus and immune reconstitution after allogeneic hematopoietic stem cell transplantation in humans: never say never again. Tissue Antigens (2012) 79:83–9. 10.1111/j.1399-0039.2011.01820.x 22220718

[35] Williams KM Hakim FT Gress RE. T cell immune reconstitution following lymphodepletion. Semin Immunol (2007) 19:318–30. 10.1016/j.smim.2007.10.004 18023361 PMC2180244

[36] Chazen GD Pereira GM LeGros G Gillis S Shevach EM. Interleukin 7 is a T-cell growth factor. Proc Natl Acad Sci U S A (1989) 86:5923–7. 10.1073/pnas.86.15.5923 2788279 PMC297743

[37] Costello R Brailly H Mallet F Mawas C Olive D. Interleukin-7 is a potent co-stimulus of the adhesion pathway involving CD2 and CD28 molecules. Immunology (1993) 80:451–7. 7904590 PMC1422227

[38] Schluns KS Kieper WC Jameson SC Lefrançois L Interleukin-7 mediates the homeostasis of naïve and memory CD8 T cells in vivo. Nat Immunol (2000) 1:426–32. 10.1038/80868 11062503

[39] Kielsen K Jordan KK Uhlving HH Pontoppidan PL Shamim Z Ifversen M T cell reconstitution in allogeneic haematopoietic stem cell transplantation: prognostic significance of plasma interleukin-7. Scand J Immunol (2015) 81:72–80. 10.1111/sji.12244 25263171

[40] Thiant S Yakoub-Agha I Magro L Trauet J Coiteux V Jouet J-P Plasma levels of IL-7 and IL-15 in the first month after myeloablative BMT are predictive biomarkers of both acute GVHD and relapse. Bone Marrow Transplant (2010) 45:1546–52. 10.1038/bmt.2010.13 20190846

[41] Dean RM Fry T Mackall C Steinberg SM Hakim F Fowler D Association of serum interleukin-7 levels with the development of acute graft-versus-host disease. J Clin Oncol (2008) 26:5735–41. 10.1200/JCO.2008.17.1314 19001329 PMC2645098

[42] Rosenberg SA Sportès C Ahmadzadeh M Fry TJ Ngo LT Schwarz SL IL-7 administration to humans leads to expansion of CD8+ and CD4+ cells but a relative decrease of CD4+ T-regulatory cells. J Immunother (2006) 29:313–9. 10.1097/01.cji.0000210386.55951.c2 16699374 PMC1473976

[43] Le Campion A Pommier A Delpoux A Stouvenel L Auffray C Martin B IL-2 and IL-7 determine the homeostatic balance between the regulatory and conventional CD4+ T cell compartments during peripheral T cell reconstitution. J Immunol (2012) 189:3339–46. 10.4049/jimmunol.1103152 22933631

[44] Heninger A-K Theil A Wilhelm C Petzold C Huebel N Kretschmer K IL-7 abrogates suppressive activity of human CD4+CD25+FOXP3+ regulatory T cells and allows expansion of alloreactive and autoreactive T cells. J Immunol (2012) 189:5649–58. 10.4049/jimmunol.1201286 23129754

[45] Lundström W Fewkes NM Mackall CL IL-7 in human health and disease. Semin Immunol (2012) 24:218–24. 10.1016/j.smim.2012.02.005 22410365 PMC3358500

[46] Jäger J Schulze C Rösner S Martin R. IL7RA haplotype-associated alterations in cellular immune function and gene expression patterns in multiple sclerosis. Genes Immun (2013) 14:453–61. 10.1038/gene.2013.40 23985573

[47] Gregory SG Schmidt S Seth P Oksenberg JR Hart J Prokop A Interleukin 7 receptor alpha chain (IL7R) shows allelic and functional association with multiple sclerosis. Nat Genet (2007) 39:1083–91. 10.1038/ng2103 17660817

[48] Crawley AM Faucher S Angel JB. Soluble IL-7R alpha (sCD127) inhibits IL-7 activity and is increased in HIV infection. J Immunol (2010) 184:4679–87. 10.4049/jimmunol.0903758 20304824

[49] Rajasuriar R Booth D Solomon A Chua K Spelman T Gouillou M Biological determinants of immune reconstitution in HIV-infected patients receiving antiretroviral therapy: the role of interleukin 7 and interleukin 7 receptor α and microbial translocation. J Infect Dis (2010) 202:1254–64. 10.1086/656369 20812848

[50] Hartling HJ Ryder LP Ullum H Ødum N Nielsen SD. Gene variation in IL-7 receptor (IL-7R) α affects IL-7R response in CD4 + T cells in HIV-infected individuals. Sci Rep (2017) 7:1–8. 10.1038/srep42036 28181541 PMC5299473

[51] Poiret T Rane L Remberger M Omazic B Gustafsson-Jernberg A Vudattu NK Reduced plasma levels of soluble interleukin-7 receptor during graft-versus-host disease (GVHD) in children and adults. BMC Immunol (2014) 15:25. 10.1186/1471-2172-15-25 24946690 PMC4074150

[52] Shamim Z Müller K Svejgaard A Poulsen LK Bodtger U Ryder LP Association between genetic polymorphisms in the human interleukin-7 receptor α-chain and inhalation allergy. Int J Immunogenet (2007) 34:149–51. 10.1111/j.1744-313X.2007.00657.x 17504502

[53] Todd JA Walker NM Cooper JD Smyth DJ Downes K Plagnol V Robust associations of four new chromosome regions from genome-wide analyses of type 1 diabetes. Nat Genet (2007) 39:857–64. 10.1038/ng2068 17554260 PMC2492393

[54] Badot V Luijten RKMAC van Roon JA Depresseux G Aydin S Van den Eynde BJ Serum soluble interleukin 7 receptor is strongly associated with lupus nephritis in patients with systemic lupus erythematosus. Ann Rheum Dis (2013) 72:453–6. 10.1136/annrheumdis-2012-202364 23264357

[55] Kreft KL Verbraak E Wierenga-Wolf AF van Meurs M Oostra BA Laman JD Decreased systemic IL-7 and soluble IL-7Rα in multiple sclerosis patients. Genes Immun (2012) 13:587–92. 10.1038/gene.2012.34 22914435

[56] Hartgring SAY van Roon JAG Wenting-van Wijk M Jacobs KMG Jahangier ZN Willis CR Elevated expression of interleukin-7 receptor in inflamed joints mediates interleukin-7-induced immune activation in rheumatoid arthritis. Arthritis Rheum (2009) 60:2595–605. 10.1002/art.24754 19714586

[57] Monti P Brigatti C Krasmann M Ziegler AG Bonifacio E Concentration and activity of the soluble form of the Interleukin-7 receptor alpha in type I diabetes identifies an interplay between hyperglycemia and immune function. Diabetes (2013) 62:2500–8. 10.2337/db12-1726 23454692 PMC3712069

[58] Lundström W Highfill S Walsh STR Beq S Morse E Kockum I Soluble IL7Rα potentiates IL-7 bioactivity and promotes autoimmunity. Proc Natl Acad Sci U S A (2013) 110:E1761–70. 10.1073/pnas.1222303110 23610432 PMC3651437

[59] Milford TAM Su RJ Francis OL Baez I Martinez SR Coats JS TSLP or IL-7 provide an IL-7Rα signal that is critical for human B lymphopoiesis. Eur J Immunol (2016) 46:2155–61. 10.1002/eji.201646307 27325567 PMC5056642

[60] Socié G Ritz J Current issues in chronic graft-versus-host disease. Blood (2014) 124:374–84. 10.1182/blood-2014-01-514752 24914139 PMC4102710

