# Supplementary material for: Donor Genotype in the Interleukin-7 Receptor α-Chain Predicts Risk of Graft-versus-Host Disease and Cytomegalovirus Infection after Allogeneic Hematopoietic Stem Cell Transplantation
Source: Front Immunol. 2018 Feb 2;9:109. doi: 10.3389/fimmu.2018.00109 (PMC5801419; doi:10.3389/fimmu.2018.00109)
Supplement: Supplementary file 1 [file table_1.PDF]

Supplementary Table. Treatment-related complications by donor IL-7Rα genotype

	Univariable model (HR (95% CI))					Multivariable model (HR (95% CI))				
	CC	CT	*P* value (CT vs. CC)	TT	*P* value (TT vs. CC)	CC	CT	*P* value (CT vs. CC)	TT	*P* value (TT vs. CC)
**Acute GVHD (grade III-IV)**	1.0	**2.03 (1.07-3.86)**	**0.031**	2.04 (0.68-6.10)	0.20	1.0	**2.02 (1.03-3.97)**	**0.040**	1.92 (0.63-5.87)	0.25
**Chronic GVHD (extensive)**	1.0	1.40 (0.95-2.07)	0.090	**2.58 (1.46-4.58)**	**0.0012**	1.0	1.31 (0.87-1.97)	0.20	**1.99 (1.09-3.64)**	**0.025**
**CMV infection**	1.0	1.31 (0.90-1.91)	0.15	**2.00 (1.10-3.65)**	**0.024**	1.0	1.30 (0.88-1.92)	0.19	**2.30 (1.24-4.27)**	**0.0083**
**EBV infection**	1.0	0.94 (0.47-1.89)	0.86	0.40 (0.05-2.96)	0.37	1.0	1.13 (0.55-2.35)	0.74	0.47 (0.06-3.35)	0.46
**Overall survival**	1.0	**1.50 (1.09-2.08)**	**0.013**	0.99 (0.50-1.98)	0.98	1.0	**1.65 (1.19-2.29)**	**0.0027**	0.90 (0.45-1.81)	0.77
**TRM**	1.0	**2.29 (1.38-3.78)**	**0.0013**	1.07 (0.40-2.90)	0.89	1.0	**2.28 (1.29-4.03)**	**0.0047**	0.56 (0.15-2.09)	0.39
**Relapse**	1.0	0.72 (0.43-1.20)	0.21	0.52 (0.16-1.65)	0.27	1.0	0.79 (0.46-1.34)	0.38	0.43 (0.12-1.47)	0.18
